# Supplementary material for: Factors associated with the willingness to provide telerehabilitation by physiotherapists treating older adults or people with neurological diseases during the COVID-19 pandemic in Sweden
Source: PLOS Digit Health. 2024 Jul 31;3(7):e0000563. doi: 10.1371/journal.pdig.0000563 (PMC11290685; doi:10.1371/journal.pdig.0000563)
Supplement: S1 File — (DOCX) [file pdig.0000563.s001.docx]

| **S1 File.** Variables and criteria used for categorization of the independent and dependent variables | | | |
| --- | --- | --- | --- |
| **Domain** | **Item description** | **Original response categories** | **Criteria for categorization** |
| *Independent variables* | | | |
| 1. **Demographic and workplace demographics** | | | |
| Sex |  | 1. Male 2. Female |  |
| Age (years) | What is your age? | 1. 20-29 2. 30-39 3. 40-49 4. 50-59 5. >59 | Responses were recategorized into  ‘20-39’ (response 1-2), ‘40-49’ or ‘>50’ (response 4-5) |
| Highest education achieved | What is your highest educational qualification within physiotherapy? | 1. Bachelor’s degree 2. Master’s degree 3. Clinical specialist 4. Doctor of philosophy | Responses were recategorized into ‘bachelor’s degree’ (response 1) or ‘graduate level or higher’ (response 2-4) |
| Work experience (years) | How many years have you worked as a physiotherapist? | 1. 1-5 years 2. 6-10 years 3. 10-19 years 4. >19 years | Responses were recategorized into:  ‘< 10 years’ (response 1-2), ‘10-19’ years or ‘>19 years’ |
| Primary patient group | Which patient group do you primarily treat? | 1. Geriatric patients 2. Neurology 3. Other | Responses 1 and 2 were included in the data set for analysis.  Response 3 was excluded from all analyses |
| Work setting | In what work setting are you primarily employed? | 1. Primary care 2. Rehabilitation center 3. Hospital (outpatient care) 4. Hospital (inpatient care) 5. University or college hospital 6. Municipal and community care | Responses were recategorized as into: ‘Primary care and rehabilitation centers’ (response 1 or 2)  ‘Hospital’ (response 3-5) or ‘Community care’ (response 6) |
| **Previous use of telerehabilitation** | | | |
| Previous use (ref. no) | Which statement about telerehabilitation best reflects your work with patients before the covid-19 pandemic? | 1. All rehabilitation was performed remotely. 2. About half of the patients were treated remotely 3. A few patients were treated remotely. 4. No patients were treated remotely | Responses 1-3 classed as having used digital tools/telerehabilitation before the COVID-19 pandemic |
| **Perception of telerehabilitation** | | | |
| Comfort | I am comfortable using digital tools (e.g., computer, tablet, or mobile application) for telerehabilitation | 1. Totally agree. 2. Agree somewhat. 3. Disagree somewhat. 4. Not correct at all | Responses 1-2 indicated a positive perception regarding digital tools/telerehabilitation. |
| Accessibility | I believe that digital tools will increase the accessibility of rehabilitation for the patients I treat |  |  |
| Patient appreciation | I think patients will appreciate telerehabilitation |  |  |
| Patient capability | Most patients I treat can use digital tools (e.g., computer, tablet, or mobile application) in their rehabilitation |  |  |
| Patient access | Most patients I treat have access to a computer, tablet, or mobile phone |  |  |
| Workplace access | My workplace has access to digital tools for telerehabilitation |  |  |
| Supportive colleagues | I perceive support from my colleagues and my boss regarding new digital tools for telerehabilitation |  |  |
| Financial reimbursement | The reimbursement system that applies to my workplace facilitates telerehabilitation with the support of digital tools |  |  |
| *Dependent variables* | | | |
| Willingness to use telerehabilitation in clinical practice | How much of your workday would you consider devoting to telerehabilitation using digital tools? | 1. Entire working day 2. Half working day 3. 1-2 hours per working day 4. A few times per week 5. Not at all | Responses 1-3 indicated a willingness to use telerehabilitation in clinical practice. |
| Provision of telerehabilitation during the COVID-19 pandemic | Which statement about telerehabilitation best reflects your work with patients during the covid-19 pandemic? | 1. All rehabilitation is performed remotely. 2. About half of the patients are treated remotely 3. A few patients are treated.   remotely   1. No patients are treated remotely. | Responses 1-3 classed as having used telerehabilitation during the COVID-19 pandemic |
