# Supplementary material for: Factors associated with the willingness to provide telerehabilitation by physiotherapists treating older adults or people with neurological diseases during the COVID-19 pandemic in Sweden
Source: PLOS Digit Health. 2024 Jul 31;3(7):e0000563. doi: 10.1371/journal.pdig.0000563 (PMC11290685; doi:10.1371/journal.pdig.0000563)
Supplement: Attachment — Submitted filename: Response to reviewers.docx [file pdig.0000563.s002.docx]

**Response to reviewers**

We would like to thank the editor and reviewers for their time and helpful feedback regarding our manuscript. Below we reply to the comments and signpost the changes in the manuscript where applicable.

Reviewer #1: I have no major issues with this manuscript.

Comment: The study aimed to identify factors associated with (i) willingness to use tele-rehabilitation and, (ii) reported use of tele-rehabilitation in clinical practice during the COVID-19 pandemic among physiotherapists (PTs) in Sweden and achieves these objectives. Although pertaining to only Sweden, the results of the study could be useful to the wider physiotherapy profession globally, in informing future work in this area. The use of digital tools is becoming more popular and as such, provides some insights into this for PTs.

The introduction is informative, and acknowledges the benefits to patients, but nothing about risks/drawbacks. I would like to see a mentioned aspects that do not benefit patients and can be exclusive, for example digital poverty. Not everyone has access and can do this type of rehabilitation. This is alluded to in the results when they refer to ”patient access"(line 246), but should be acknowledged.

Response:

Thank you for your comment, we agree that telerehabilitation and digital healthcare can also be very excluding. We have added a paragraph in the methods section which addresses this and provides some references for support (line 103-112).

Comment: The methods are generally well written. It is helpful to refer to the previous publication of the recruitment and study data collection methods which have been previously published. Figures and tables are clear and appropriate. Results are clear.

Response:

Thank you. We have now included more references to the previously published publication in the methods section (lines 165 and 182).

Comment: In the discussion, it is interesting that the willingness to use digital tools, did not include concerns for patient safety from PTs, but patient appreciation. Was patient safety mentioned at all in the questionnaire; could this be discussed as in previous literature.

Response:

Thank you for your insightful comment. Unfortunately, we did not include a question about safety in the questionnaire, but we agree it would have been interesting to include. We have now included a paragraph in the discussion about this though (line 333-340).

Conclusion is appropriate.

Limitations are appropriate.

Reviewer #2:

Comment: Clinically relevant paper finding that among physiotherapists in Sweden, willingness to use tele-rehabilitation is largely associated with personal attitudes towards it, whereas reported use of tele-rehabilitation appears to be related to organisational factors. These findings could be used to shape future implementation of tele-rehabilitation practices in geriatric and neurorehabilitation in Sweden and contribute to the broader understanding of tele-rehabilitation among physiotherapists across different contexts.

Response:

Thank you for your kind comments. We are happy to hear you see our findings as valuable for understanding the future of implementation of telerehabilitation.

Reviewer #3:

A well written manuscript with novel and interesting findings presented. Statistical analyses are performed appropriately and rigourously with figures and tables presented to support claims. Conclusions are supported by the data.

Comment: In response to Q3, authors have specified that data is not able to be publicly shared due to participant privacy, but data will be made available upon request.

Response:

Thank you for pointing out this inconsistency. We have now amended the data statement (line 419-423).
